# Effect of urine reflex culturing on rates of cultures and infections in acute and long-term care

**DOI:** 10.1186/s13756-020-00762-1

**Published:** 2020-06-29

**Authors:** Chelsea S. Lynch, Andrea Appleby-Sigler, Jacqueline T. Bork, Rohini Davé, Kathy Agnes, Molly Sanikop, Doris Heath, Arlene F. Clark, Kimberly Claeys, Min Zhan, Daniel J. Morgan

**Affiliations:** 1 Department of Infection Control, VA Maryland Health Care System, Baltimore, MD USA; 2 Microbiology Department, VA Maryland Health Care System, Baltimore, MD USA; 3 Department of Infectious Diseases, VA Maryland Health Care System, Baltimore, MD USA; 4 Department of Medicine, University of Maryland, Baltimore, MD USA; 5 Department of Pharmacy, VA Maryland Health Care System, Baltimore, MD USA; 6 Department of Pharmacy Practice and Science, University of Maryland, Baltimore, MD USA; 7 Department of Epidemiology and Public Health, University of Maryland, 10 S, Pine St. MSTF 334, Baltimore, MD 21201 USA

**Keywords:** Stewardship, Urinary tract infections, Diagnostic microbiology

## Abstract

**Background:**

Urine cultures are often positive in the absence of a urinary tract infection (UTI). Pyuria is generally considered necessary to diagnose a UTI.

**Problem:**

Urine cultures are often positive in the absence of UTI leading to unnecessary antibiotics.

**Methods:**

Quasi-experimental pre-post study of all patient urine cultures ordered in a VA acute care hospital, emergency department (ED), and two long-term care (LTC) facilities from August 2016 to August 2018. Urine cultures performed per 100 days were compared pre- (August 2016 to July 2017) versus post-intervention (August 2017 to August 2018) using interrupted time series negative binomial regression.

**Intervention:**

We examined whether reflexing to urine culture only if a urinalysis (UA) found greater than 10 WBC/hpf decreased urine culturing.

**Results:**

In acute-care, reflex culturing resulted in a 39% time series regression analysis adjusted decrease in the rate of cultures performed (pre-intervention, 3.6 cultures/100 days vs. Post-intervention, 1.8 cultures/100 days, *p* < 0.001). Pre-intervention, 29% (4/14) of Catheter-associated UTI (CAUTI) would not have been reported if reflex culturing was employed. In the ED, reflex culturing was associated with a 38% (*p* = 0.0015) regression analysis adjusted decrease in cultures, from 5.4/100 visits to 3.3/100 visits. In LTC, there was a small absolute, but regression analysis adjusted increase of 89% (*p* = 0.0018) in rates from (0.4/100 days to 0.5/100 days).

**Conclusion:**

In acute care and ED, urine reflex culturing decreased the number of urine cultures performed. A small absolute increase was seen between pre-post time periods in LTC. Reflex testing generally decreases cultures and may lead to more accurate diagnoses of CAUTI.

## Introduction

Urinary tract infections (UTI) are one of the most common healthcare-associated infections, accounting for more than 12% of infections reported by acute care hospitals [1] and 20% of infections reported by long-term care (LTC) facilities [2].

### Problem description

Urine cultures are frequently ordered as a part of a generalized workup for non-specific symptoms (e.g. fevers without traditional UTI symptoms such as dysuria and frequency) [3]. This often results in false-positive urine culture results [4]. Clinically, this is known as asymptomatic bacteriuria (ASB). False-positive urine cultures are associated with unnecessary antibiotic treatment of ASB and elevated catheter-associated UTI (CAUTI) rates [5].

### Available knowledge

Reflex urine reflex culturing, defined as only performing a urine culture if the preceding UA showed pyuria [6–9]. may improve unnecessary use of antibiotics for false-positive urine cultures.

### Rationale

Urine reflex culturing has been shown to decrease the rate of urine cultures performed in adult intensive care units but the benefit in other settings is unknown [9, 10].

### Specific aims

We evaluated the impact of implementation of urine reflex culturing across a healthcare system including patient care in acute care, the emergency department (ED), and (LTC) facilities.

## Methods

### Context

This was a quasi-experimental study at an integrated healthcare system. The VA Maryland Health Care System (VAMHCS) is comprised of three medical centers operating approximately 727 inpatient beds (acute care and LTC) as well as an emergency department with 22 beds and six outpatient clinics throughout Maryland. The study received ethics review and approval from the University of Maryland Baltimore Institutional Review Board.

An internet-based, data mining surveillance tool (TheraDoc,DSS inc.) was used to identify all urine cultures ordered from August 2016 to August 2018 based on collection location. Acute care included five inpatient units (three intensive care units, one medical unit, and one surgical unit). The ED included all emergency room visits. LTC included all eight LTC units in two facilities (including rehabilitation patients and hospice patients). Patients with urine testing in the ED would not be included in the analysis of acute-care unless they had a separate urine test done as an inpatient. Multiple admissions per patient were included in analysis.

### Intervention

Urine reflex culturing criterion was a urinalysis with > 10 white blood cells per high power field (WBC/hpf). This was instituted throughout the system in August 2017.

### Study of the interventions

Each urine culture result was categorized as negative, positive < 100,000 colony forming units per milliliter (CFU/ml), or positive ≥100,000 CFU/ml. No urine cultures were excluded.

CAUTIs for acute care and LTC were reviewed from August 2015 to August 2018 using Centers for Disease Control and Prevention’s National Healthcare Safety Network (NHSN) criteria. For acute care, [1] the criteria was a positive urine culture with no more than two species of organisms with at least one being a bacterium of ≥100,000 CFU/ml obtained with an indwelling catheter in place for at least two calendar days (or removed within two calendar days of indwelling catheter removal) and at least one documented sign or symptom (fever > 38.0 °C; suprapubic tenderness; costovertebral angle pain or tenderness; urinary urgency; urinary frequency; or dysuria).

For LTC, [2] the criteria was a positive urine culture with no more than two species of organisms with at least one being a bacterium of ≥100,000 CFU/ml obtained with an indwelling catheter in place for at least two calendar days (or removed within two calendar days of indwelling catheter removal) and at least one documented sign or symptom (fever > 37.8 °C; rigors; new onset of hypotension; new onset of confusion/functional decline and leukocytosis; new or marked increase in suprapubic tenderness; new or marked increase in costovertebral angle pain or tenderness; acute pain, swelling, or tenderness of the testes, epididymis, or prostate; purulent discharge from around catheter insertion site).

### Measures

Rate of urine culture.

### Analysis

Comparisons pre- and post-intervention were made using time series regression analysis. Negative binomial regression was used to assess immediate changes and trends in the number of urine cultures performed. A *p*-value of < 0.05 was considered statistically significant. All analysis was competed using SAS v 9.4 (SAS Institute, Cary, NC).

### Ethical considerations

This description of a patient safety initiative was considered non-human subjects research.

## Results

### Acute care

**Fig. 1:**
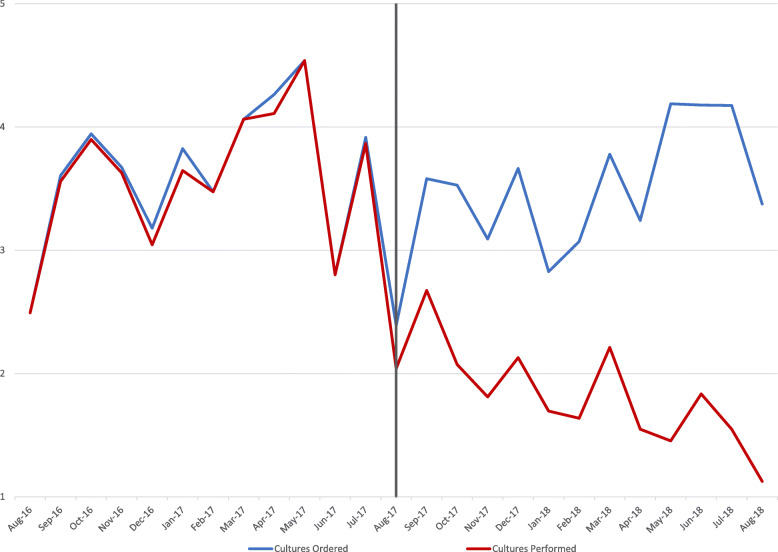
Acute Care Urine Cultures Ordered vs. Urine Cultures Performed per 100 Days

In the pre-intervention period, 908 urine cultures were ordered, and 894 urine cultures were performed, a rate of 3.58 cultures per 100 days. In the post-intervention period, 965 urine cultures were ordered, and 507 urine cultures were performed, a rate of 1.82 cultures performed per 100 days. With the institution of urine reflex culturing, there was an regression analysis adjusted immediate 39% decrease in the rate of cultures performed (*p* < 0.0001) with a monthly decrease of 6% through the post-intervention period (*p* = 0.0003). Urine reflex culturing cancelled 45% of urine cultures ordered in the post-intervention period, with 458 cultures cancelled (See Fig. 1). Applying urine reflex culturing during the pre-implementation period would have resulted in 29% fewer reported CAUTIs.

### Emergency department (ED)

**Fig. 2:**
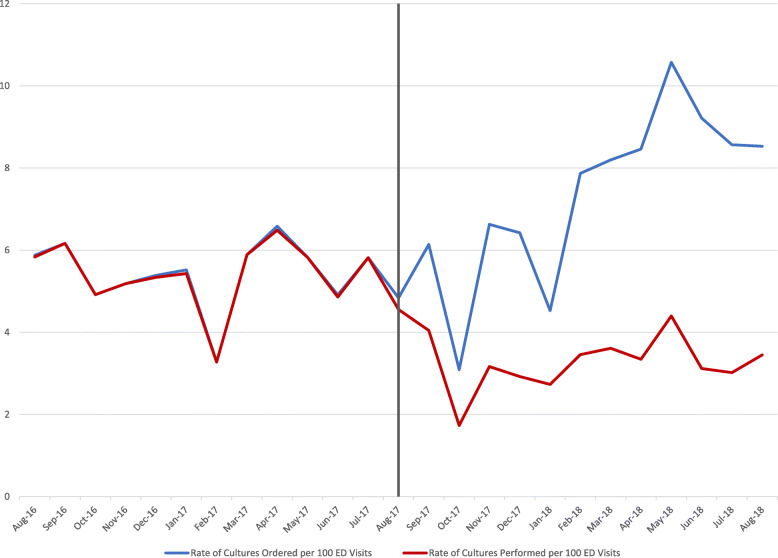
Emergency Department Urine Cultures Ordered vs. Urine Cultures Performed per 100 ED Visits

In the pre-intervention period, 1400 urine cultures were ordered, and 1393 urine cultures were performed, a rate of 5.44 cultures performed per 100 ED visits. In the post-intervention period, 1959 urine cultures were ordered, and 917 urine cultures were performed (1042 cancelled), a rate of 3.34 cultures performed per 100 ED visits. With the institution of urine reflex culturing, there was an regression analysis adjusted immediate 38% decrease in the rate of cultures performed (*p* = 0.0015) with similar monthly rates of 0.12% through the post-intervention period (*p* = 0.9541). Urine reflex culturing cancelled 51% of urine cultured ordered in the post-intervention period (see Fig. 2).

### Long-term care (LTC)

**Fig. 3:**
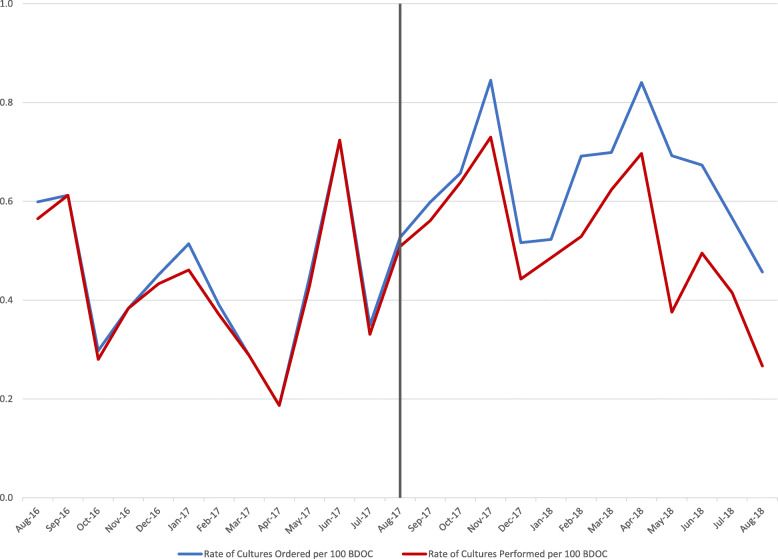
Long-Term Care Urine Cultures Ordered vs. Urine Cultures Performed per 100 days

In the pre-intervention period, 267 urine cultures were ordered, and 257 urine cultures were performed, a rate of 0.41 cultures performed per 100 days. In the post-intervention period, 432 urine cultures were ordered, and 354 urine cultures were performed, a rate of 0.52 cultures performed per 100 days. With the institution of urine reflex culturing, there was a regression analysis adjusted immediate 89% increase in cultures performed (*p* < 0.0001) and the monthly trend through the post-intervention period stayed the same as in pre-intervention period (*p* = 0.83). However, urine reflex culturing cancelled 16% of urine cultures ordered in the post-intervention period (see Fig. 3).

### Catheter-associated urinary tract infections (CAUTI)

In acute care, from August 2015 to July 2017, there were 14 CAUTI with a rate of 1.82 infections per 1000 catheter days. Urine reflex culturing would have canceled the triggering urine cultures for 4 (29%) of those CAUTI, decreasing the rate to 1.30 infections per 1000 catheter days. In the post-intervention period, there were 6 CAUTI with a rate of 1.64 infections per 1000 catheter days.

In LTC, from August 2015 to July 2017, there were 13 CAUTI with a rate of 0.99 infections per 1000 catheter days. Urine reflex culturing would have canceled the triggering urine cultures for 1 (8%) of those CAUTI, decreasing the rate to 0.91 infections per 1000 catheter days. In the post-intervention period there were 6 CAUTI with a rate of 1.60 infections per 1000 catheter days.

## Discussion

### Summary

Urine reflex culturing decreased the rate of urine cultures. Urine reflex culturing caused the greatest decrease on urine culturing in acute care and the emergency department but was associated with a small absolute *increase* in urine cultures long-term care. Although rates of CAUTI did not change with reflex culturing, almost 1/3 of CAUTIs occurred in patients without pyuria in the pre-period in acute care and were likely mis-diagnosed.

### Interpretation

Many urine cultures were cancelled due to reflex testing in acute care and ED with many fewer cancelled in LTC. This is likely due to the differences in patient populations with more chronic pyuria among LTC patients. A higher urine reflex culturing cutoff for pyuria (e.g. > 25 WBC/hpf or > 50 WBC/hpf) may have resulted in better performance among this population but this is unknown. The cutoff of > 10 WBC/hpf was chosen as it is the most common definition of pyuria, but it is unclear if it is the most effective cutoff for optimal urine culturing.

Urine reflex culturing was well accepted by the Microbiology Laboratory where it resulted in cost savings due to the decreased number of urine cultures performed. Additionally, the intervention was accepted by clinicians who interpreted cancelation of urine cultures due to lack of pyuria as negative results. This likely relates to the extensive education to the clinicians about the intervention before it was implemented.

### Limitations

While urine reflex culturing reduced urine cultures performed, it mostly reduced cultures that would have been negative. It also only partially addressed bacteriuria and ASB, as pyuria was often present in patients with ASB. The potential downstream clinical consequences of urine reflex testing, in particular, antibiotic therapy, have not been fully elucidated. It is notable that using reflex culturing during the pre-period would have resulted in reporting 29% fewer CAUTIs in acute care. Patients could be included more than once if they had multiple admissions. We did not evaluate patient characteristics before and after intervention, although there were no broad changes to the services provided at our facilities. The appropriate use of urine cultures still requires careful clinical judgment to avoid ordering when patients are asymptomatic and interpreted carefully to avoid unnecessary antibiotics.

## Conclusion

We found that urine reflex culturing was safe and easy to implement in acute care, ED care, and LTC and led to a nearly 40% decrease in cultures in the hospital. This intervention likely decreased the number of patients treated for ASB, and misidentification of CAUTIs, while reducing microbiology costs.

## Data Availability

There are legal restrictions on sharing a de-identified data set from Veterans Affairs (VA) because of the inclusion of report data and other details that make it extremely difficult to de-identify. VA has generally not allowed release of de-identified data because they are usually re-identifiable, at least in part. If data are requested, they may apply for data access by submitting an approved IRB protocol and VA Research and Development Committee Approval letter to the Veterans Informatics and Computing Infrastructure. Queries can be directed to VINCI@va.gov.

## References

[1] National Healthcare Safety Network (NHSN). Urinary Tract Infection (Catheter-Associated Urinary Tract Infection [CAUTI] and Non-Catheter-Associated Urinary Tract Infection [UTI]) and Other Urinary Symptom Infection [USI]) Events. [Internet]. Patient Safety Component Manual. Center for Disease Control and Prevention; 2019 [cited 2019 Aug 1]. Available from: https://www.cdc.gov/nhsn/pdfs/pscmanual/pcsmanual_current.pdf.

[2] National Healthcare Safety Network (NHSN). Healthcare-Associated Infection Surveillance Protocol for Urinary Tract Infection Events for Long-Term Care Facilities [Internet]. Center for Disease Control and Prevention.NHSN Long-Term Care Facility Component; [cited 2019 Aug 1]. Available from: https://www.cdc.gov/nhsn/PDFs/LTC/LTCF-UTI-protocol-current.pdf.

[3] Drekonja DM, Gnadt C, Kuskowski MA, Johnson JR (2014). Urine cultures among hospitalized veterans: casting too broad a net?. Infect Control Hosp Epidemiol.

[4] Nicolle LE, Gupta K, Bradley SF, Colgan R, DeMuri GP, Drekonja D (2019). Clinical practice guideline for the Management of Asymptomatic Bacteriuria: 2019 update by the infectious diseases Society of Americaa. Clin Infect Dis.

[5] Morgan DJ, Malani P, Diekema DJ (2017). Diagnostic stewardship—leveraging the laboratory to improve antimicrobial use. JAMA..

[6] Jones CW, Culbreath KD, Mehrotra A, Gilligan PH (2014). Reflect urine culture cancellation in the emergency department. J Emergency Med.

[7] Stagg A, Lutz H, Kirpalaney S, Matelski JJ, Kaufman A, Leis J (2018). Impact of two-step urine culture ordering in the emergency department: a time series analysis. BMJ Qual Saf.

[8] Epstein L, Edwards JR, Halpin AL, Preas MA, Blythe D, Harris AD (2016). Evaluation of a novel intervention to reduce unnecessary urine cultures in intensive care units at a tertiary Care Hospital in Maryland, 2011–2014. Infect Control Hosp Epidemiol.

[9] Sarg M, Waldrop GE, Beier MA, Heil EL, Thom KA, Preas MA (2016). Impact of changes in urine culture ordering practice on antimicrobial utilization in intensive care units at an Academic Medical Center. Infect Control Hosp Epidemiol.

[10] Claeys KC, Blanco N, Morgan DJ, Leekha S, Sullivan KV (2019). Advances and challenges in the diagnosis and treatment of urinary tract infections: the need for diagnostic stewardship. Curr Infect Dis Rep.

